# Sensehacking the guest’s multisensory hotel experience

**DOI:** 10.3389/fpsyg.2022.1014818

**Published:** 2022-12-19

**Authors:** Charles Spence

**Affiliations:** Department of Experimental Psychology, University of Oxford, Oxford, United Kingdom

**Keywords:** sensory marketing, hotel, sensehacking, multisensory, experiential, biophilia

## Abstract

This narrative review discusses the literature on contemporary sensory marketing as it applies to hotel design. The role of each of the guest’s senses in the different stages of the customer journey are highlighted, and the functional benefits (to the guest’s multisensory experience), and likely commercial gains, of engaging more effectively with the guest’s non-visual senses, both individually, and in combination, are reviewed. While the visual elements of hotel design are undoubtedly important, the hotelier neglects the non-visual senses at their peril, given the negative effect of poor design on the customers’ overall multisensory experience (and ratings). A number of the crossmodal effects and multisensory interactions that have been suggested to modulate the guest’s experience of hotels (and resorts) are discussed. Mention is also made of the nature effect/biophilic design and how it is increasingly being incorporated in total design to help deliver on guest/customer well-being; the latter is a theme that has grown rapidly in relevance for those working in the hospitality sector. Taken together, there are numerous opportunities for hotel managers to ‘sensehack’ their guests’ multisensory experiences through environmental psychology The originality of this review stems from the analysis of the hierarchy of the guest’s senses and an explanation of how multisensory interactions affect sensory marketing in the design of hotel experiences for guests.

## Introduction

In recent years, many hotels have recognized the need to ‘up their game’ in terms of delivering the ‘experience’ to their guests, given the rise of Airbnb, etc. (e.g., see Xu and Chan, 2010; TMI Spy Hotels and Leisure, 2015; Hunt, 2016). It is, though, interesting to note that in his classic paper, ‘Atmospherics as a marketing tool’, Kotler (1974) barely mentions hotels at all when discussing the importance of atmospherics to the total experience of retail (even though he discusses travel agencies and the psychiatrist’s office). In fact, the sole exception comes when the famous marketer notes that “Management must next identify the major atmospheric variables that are available to produce the desired customer awareness and reaction. It must consider how sight, sound, scent, and texture can each contribute to attaining the desired total effect. For example, the Hampshire House in Chicago caters to persons who want the sense of staying at “the Grand Hotel.” Consequently, the hotel has been atmospherized in a way which thoroughly suggests luxurious surroundings and service. “The rustle of silk. The smell of leather luggage. Palms in regal pots…” (Kotler, 1974, p. 62).^1^

Hotels also fail to attract much of Pine and Gilmore’s (1998, 1999) attention in their influential discussion of the importance of the ‘experience economy’, though there has been a growth in experiential marketing in the high-end hotel sector (see also Gilmore and Pine, 2002a, pp. 88–89; Gilmore and Pine, 2002b), as hoteliers try to ensure that their guests leave having had an unforgettable experience (Schmitt, 1999; see also Nurminen, 2003; Thomson, 2010; Vardarı and Arapı, 2017). As we will see later, part of the way in which this can be delivered is by offering extraordinary dining and drinking experiences (e.g., Hanefors and Mossberg, 2003; Spence, 2017; Kiatkawsin and Han, 2019). Indeed, a growing number of hotels are now pandering to their guests’ desire to engage in ‘Sensploration’ (see Gray, 2019).^2^ It is certainly intriguing to see how innovative sensory solutions in this space have often been sourced from the ideas originally developed in the context of high-end dining (see Aroche, 2015; Spence, 2017).

Delivering on the promise of a luxury or premium experience/stay typically necessitates intelligently engaging the guest’s non-visual senses (Wiedmann et al., 2013). In the sections that follow, a number of innovative sensory interventions will be reviewed. That said, while several authors have highlighted the importance of multisensory stimulation to the delivery of a luxury experience (Wiedmann et al., 2016), oftentimes few actionable insights are offered, other than, perhaps, to consider a greater use of scent (Crouse, 2010; Sklavos, 2020), and/or to personalize the guest’ multisensory experience (see also Gilmore and Pine, 2002a; Shahid and Paul, 2022). For instance, Wiedmann et al. (2016) describe “a multisensory approach that promises the ultimate luxurious experience, sensorial stimulation of the guests’ sight, taste, hearing, smell, and touch is provided by the use of appropriate colors, tunes, scents, flavours and materials.”

That said, I surely cannot be the only one who is sick of being asked: “How was the experience?” Indeed, some commentators have, in recent years, suggested that the ‘experience economy’ (Pine and Gilmore, 1998, 1999) may perhaps have had its day. The multisensory atmosphere undoubtedly affects the hotel guests’ experience, though often it does so without their necessarily realizing. However, one of the key challenges is knowing how to measure the guest’s experience without necessarily having to rely on all those tiresome questionnaires (Walls et al., 2011). Ultimately, the hotelier needs to understand more about the role of sensory stimulation in their guests’ overall satisfaction and return patronage decisions (Carbone, 2004; Meyer and Schwager, 2007; Ogle, 2009; Walls, 2013). What is more, they also need to move beyond the dominant visual sense in order to think about the total multisensory experience and, in particular, how to effectively stimulate the guest’s more emotional senses (namely, touch, smell, and taste).

Of course, functional/business hotels are very different in terms of the multisensory propositions that they offer destination/leisure resorts. That said, regardless of the clientele, there has undoubtedly been an increasing focus on guest wellness/well-being in recent years (Hill, 2021); Part of the solution in this space may well be linked to the ‘nature effect’ (Williams, 2017; Spence, 2021), a theme that I will return to later in this review. This narrative historical review (see Ferrari, 2015; Furley and Goldschmied, 2021, on the narrative style of review) highlights a number of the ways in which the hotel guest’s experience can be ‘sensehacked’. Sensehacking refers to the use of multisensory design to help enhance the customer’s social, cognitive, and/or emotional well-being. Sensehacking is built on a growing cognitive neuroscience understanding of the ubiquitous nature of multisensory interactions, and an acknowledgement that the senses often affect people (e.g., hotel guests) without their necessarily being aware of it.

## Multisensory matters in hospitality

### The sensory hierarchy of the guest experience

Humans are visually dominant (Heilig, 1992; Gallace et al., 2012; Hutmacher, 2019). As such, hoteliers neglect their guests’ non-visual senses at their peril. Consider here only how complaints from hotel guests often center on noise (e.g., among those guests who are trying to sleep, as presumably most guests do at some point during their stay; Sharkey, 2007). Similarly, a bad smell can exert a particularly negative influence over the guests’ impressions of an establishment (Pacelle, 1992).^3^ According to a 2013 analysis of more than 2.5 million guest reviews of 5,683 hotels in 20 major tourist destinations around the world (by ReviewPro), noise topped the list of the most common complaints in online hotel reviews. Complaints about elevators and smells ranked second and third, respectively (Stephens, 2013). Negative comments about the air conditioning and heating systems should also be noted, since they were highlighted in twice as many reviews as issues with the internet/wifi. Meanwhile, a J.D. Power and Associates guest satisfaction survey found that customers rated noise as their top concern in every hotel segment except luxury. No wonder that building quieter hotels is such a booming business (see Sharkey, 2007; Spence, 2020a).

According to a more recent analysis of the data from a hospitality ticketing app, hotel guests’ top five complaints concern temperature, with rooms being too hot/cold (mentioned by 24% of complainants); an inability to access the wifi (14%); “I can hear too much noise in my room.” (11%); “I found a ___, my room is not clean!” (10%); and poor service (9%; see Zaldivar, 2017; Wood, 2019). Notice here how three out of the top five complaints are based on the senses (see also Suh et al., 2015). In fact, problems with touch (temperature) and noise (sound) together accounted for 35% of all guest complaints as compared to just 10% of complaints being related to cleanliness (which one might consider to be a primarily visual issue).

Ogle (2009), p. 159 quizzed eight general managers of deluxe hotels (situated primarily in the Asia Pacific region) concerning the importance of the senses to their guests’ overall experience. Their responses suggested that they considered ‘sight’ to be the dominant sense, followed closely by ‘smell’. Further analysis of the hoteliers’ responses revealed that contemporary guestrooms appear to be primarily designed to be pleasing to the eye and, to a lesser extent, to the sense of touch with little, if any, consideration being given over to how they smell. This despite the latter being the second most important determinant of the positive first and lasting impression for guests. As one hotelier put it: “Strange as it may seem, I believe that the first impression of a room is made by the smell of the room as the guest enters. Once an olfactory impression of a room is made, the other senses come into play. A guest stepping into a smelly, stale room, for example, tobacco smoke, will not notice the beautiful flowers or view or anything else until the smell is removed.” (quoted in Ogle, 2009, p. 167). Stimulating the sense of smell is also important in the context of enhancing people’s sense of well-being (see Spence, 2020c, for a review).

Many people report experiencing the negative effects of sensory overload (Malhotra, 1984), and this can be linked to the growing appeal of ‘digital detox’ (Prochnik, 2011; Goodin, 2017; Bridge, 2018; Matthews, 2019). In fact, it sometimes seems as though what many people really crave when they go away is a holiday/break from all the stimulation provided by their digital technology (especially from work-related emails/messages; see also Bowles, 2019; Bridge and Knowles, 2019). Often, though, a closer inspection of such cases suggests that the real problem may not be one of sensory overload, as much as sensory imbalance (see Spence, 2002; Spence, 2021a). In particular, the higher rational senses of sight and sound would appear to be an overstimulation (often by digital technology) masking the potentially detrimental effects of an understimulation of the more emotional senses (of smell, touch, and taste; see Spence, 2002). Tiffany Field (2001) has long highlighted the ‘touch hunger’ that she believes to be prevalent in contemporary society. Bear in mind here only the fact that the tactile receptors embedded in the skin constitute the body’s largest sense, accounting for something like 16–18% of body mass (Montagu, 1971; Gallace and Spence, 2014). The rise in spa treatments would seem to be one of the ways in which people’s need for touch is increasingly being met (Spence, 2002; Levere, 2003).

While the possibility of managing the sonic elements of the guest’s experience have long been offered by the likes of the Muzak corporation (Sterne, 1997; Lanza, 2004; Prochnik, 2011), recent decades have witnessed the arrival of various solutions to the controlled delivery of ambient scent. These include the deliberate dispersal of different scents in various locations within a venue and/or at different times of the day/year (Spence, 2021c). This matches the approach adopted by the Adam & Eve Hotel in its Antalya Belek resort in Turkey.^4^ The guests in this venue are exposed to very different styles of music in each of the hotel’s public rooms/spaces. In this case, the sonic approach is deliberately designed to accommodate the hotel guests’ varied cultural backgrounds (Vardarı and Arapı, 2017; *cf.* Park et al., 2010). Meanwhile, at Sofitel’s The Grand Hotel in Amsterdam (which reopened in September 2010), and which is positioned as a super luxury hotel, different atmospheres were created in different sections of the hotel; In this case, the zoning is signaled as much by the very different visual design/color schemes as by anything else (see Derval, 2010, pp. 59–61; see also Yu et al., 2020).

According to Wiedmann et al. (2016), the Burj Khalife hotel plays Zen ambient relaxing music or Arabic sounds. In this case, the sonic backdrop obviously links to the hotel’s physical situation, as well as potentially helping the guests to relax (i.e., delivering a functional sensory benefit). Importantly, it is only by congruently stimulating the guest’s senses (e.g., by means of scent, sound, and colour; Errajaa et al., 2018) that the desired effect (be it is terms of relaxation, approach, or arousal can be achieved; see Spence, 2021a; though see Morrin and Chebat, 2005; Fenko and Loock, 2014; Sklavos, 2020, for some of the challenges associated with deliberately stimulating multiple senses in public spaces).

### From stimulating multiple senses to delivering effective multisensory stimulation

One of the problems with much of the sensory marketing literature, as in Kotler’s (1974) seminal review, is the sense-by-sense approach that so many have adopted. There is seemingly little awareness of just how much the senses interact with one another in delivering the total multisensory effect or experience. As Heschong (1979), p. 29 put it a little over four decades ago: “Since each sense contributes a slightly different perception of the world, the more senses involved in a particular experience, the fuller, the rounder, the experience becomes. If sight allows for a three-dimensional world, then each other sense contributes at least one, if not more, additional dimensions. The most vivid, most powerful experiences are those involving all of the senses at once.” Contemporary cognitive neuroscience reveals how multisensory crossmodal interactions are, in fact, the norm, rather than the exception (see Spence, 2020d). That being said, only two pages of Krishna’s (2010, pp. 371–372) edited volume mentions multisensory matters directly. The ubiquity of these crossmodal/multisensory interactions in everyday life has also been missed by much of the well-being literature (e.g., see de Vries, 1997).

To give just a few examples of the ubiquity of such crossmodal effects, note only how pleasant scents can be used to help to enhance the perceived softness and whiteness of the hotel’s towels (see Vickers and Spence, 2007; Spence, 2022a). Meanwhile, the use of a citrus scent has been shown to help to make a space look cleaner (Holland et al., 2005), while at the same time also nudging people toward leaving less mess (De Lange et al., 2012).^5^ The presence of a pleasant ambient fragrance may exert a positive influence on social interaction (Zemke and Shoemaker, 2007; Zemke and Shoemaker, 2008). Similarly, it turns out that thermal comfort is not determined only by the ambient temperature/humidity of a space, but also by the colour/lighting (Tsushima et al., 2020; see Spence, 2020b, for a review). Furthermore, it has been suggested by practitioners that lighting levels (e.g., in the hotel’s corridors) can even bias how loudly guests speak. As such, the latter can potentially be encouraged to keep the noise levels down simply by adjusting the lighting. As Sarah Simpson, Head of Product for Premier Inn notes: “Warm lighting in our corridors is used to subconsciously encourage our guests to speak in quieter tones, thus reducing disturbances to other customers who may already be asleep.” (quoted in Thornhill, 2015). Once again, notice the crossmodal sensory solution designed to help address one of the hotel guest’s most common complaints (namely concerning noise). Moving things in the opposite direction, the use of hard flooring (e.g., such as ceramic tiles at the bar) can help generate noise and thus encourage a more sociable environment (Thornhill, 2015; Masters, 2022; though see Bravo-Moncayo et al., 2020, on the potentially detrimental impact of noise on tasting experiences).

As well as trying to deliver the optimal multisensory experience at the various stages of the hotel guest’s customer journey, another relevant question that more hoteliers ought to be asking themselves here is how to prolong the multisensory experience, or rather the guest’s memory of that experience, especially their memory of the most positive elements (this is known as ‘Sticktion’; LaTour and Carbone, 2014). While the technical definition of ‘Sticktion’ refers to “the static friction that needs to be overcome to enable relative motion of stationary objects in contact,” the term is often used to refer to those things that stick in memory, and hence are not easily forgotten (given the predictable fallibility of human memory). The main point here is that positive customer experiences should be optimized for memory.

## ‘Sticktion’: Creating memorable guest experiences

The guests’ experience on arriving at a hotel (i.e., when checking in at reception) is likely to leave a lasting (and possibly disproportionately important) impression on their overall memory of their visit/stay. For example, when I think back to the last time that I visited one of Miami’s famous landmark hotels on South Beach for a trade conference in 2017, my abiding memory was the endless queue snaking back from reception on my arrival. Presumably, in this case, I turned up shortly after a busload or two of guests (an unpredictable occurrence in other words). And yet, even five years later, it is this negative first impression that stands out in your author’s memory over-and-above the scent of rooms, the softness of the towels, the view from the balcony, etc. In one intriguing example of the functional use of scent to help address such problems, the Miami Dadeland Marriott Hotel installed a $12 k machine that releases floral and citrus scents that had been tailored to the hotel’s Central and South American clientele (see Pacelle, 1992). In this case, the desired effect was to alleviate the stress of the guests, many of whom arrive somewhat frazzled after a long flight (not to mention having to navigate through US Customs; as was, in fact, the case for me as well in the above-mentioned anecdote). Aromatherapy has been described as ‘olfactory Muzak’. It might also be considered as delivering an olfactory version of the ‘nature effect’ (see Spence, 2021b; see also Bentley et al., 2022).

For several years now, The Hilton Doubletree has been scenting the lobbies of their hotels with the smell of freshly-baked cookies. According to Hoppough (2006), the chocolate-chip-cookie scent originates not from the cookies themselves (even though the chain hands out 65,000 every day to arriving guests),^6^ but from a fragrancing device provided by ScentAir. It does not seem unreasonable, therefore, to consider whether this sweet scent might also help to relieve any tensions that the guest may have when they arrive. In fact, one can almost think of it as a signature scent for the hotel chain (see Wiedmann et al., 2016; Minsky et al., 2018).

A few years ago, one of the budget hotel chains here in the UK experimented with the release of breakfast aromas at reception in the morning in the hope of encouraging/nudging a few more of their guests to pay for this extra before they left. Along very similar lines, the Hard Rock Hotel in Orlando released a waffle-cone and sugar-cookie scent into the atmosphere that successfully lured visitors to an out-of-the-way ice cream shop, where sales jumped 45% in the first six months after the scenting device was installed (see Hoppough, 2006). In this case, notice how scent was being used to help promote sales (i.e., for anticipated commercial gain) rather than necessarily to improve the guest’s experience. It should, though, be remembered that unsuccessful uses of scent are less likely to be reported on (see Spence, 2015b). Meanwhile, Westin Hotels & Resorts, uses a white tea fragrance in its 127 properties, using a similar ScentAir Technologies delivery system (Hoppough, 2006). Intriguingly, the chain have since monetized the scent by selling white tea-scented candles ($36) and fragrance diffusers (at $65 in 2006) to their guests According to Sue A. Brush, senior vice president of Westin, they were hoping for sales of $750,000 in the first year alone (see Hoppough, 2006).

Delivering a memorable multisensory experience, i.e., one that really engages the guests’ senses, means more than merely just removing desks from the guests’ rooms (given that so many Millennials apparently prefer to work from bed rather than sitting at a table), or replacing the angular (and oftentimes rather intimidating) reception desk with something rounder and more approachable (see Thornhill, 2015). Note here only how a growing body of research now shows that people find environments with a round floorplan, or furniture, more approachable than angular spaces/forms (see Dazkir and Read, 2012; Vartanian et al., 2013; Cotter et al., 2017).

While it is easy to imagine that the guests’ memory for experiences, such as a hotel stay, is simply a weaker (i.e., somewhat faded) version of the actual sensory/perceptual experience at the time it occurred (Knutson et al., 2009), this is emphatically not the case. In fact, the mind has been shown to play systematic tricks on us all. That is, we predictably remember certain elements of our experiences better than others. According to the memory literature, people are more likely to remember the start of an experience as well as perhaps how it ends. The highlight/low-point of a guest’s journey, or experience, will likely also stick in their memory much better than long periods where nothing much happens (e.g., as when the guest is simply lounging around in their room, say). And, of course, the many hours of sleep likely leave little trace in a guest’s memory though, that said, problems with getting to sleep/sleep quality may well be remembered. According to estimates, the average guest apparently spends four waking hours per day at a property (Marsan, 1999), the majority of the rest of the time is spent in their room while sleeping (or at least trying to).

The ‘experience engineers’ who have studied people’s memories for their experience of mainstream restaurants find that our recollections rarely revolve around the food. Just take the following example to illustrate the idea. When 125 customers who had eaten at a branch of the UK chain Pizza Hut were quizzed a week after their visit, it turned out that the enthusiasm of the opening greeting with the restaurant staff – the energy and warmth with which the employees introduced themselves – was the single most salient memory for most of them, not the thickness of the crust, nor the range of toppings, etc. (LaTour and Carbone, 2014). What was also important was how long it took to be acknowledged by a member of staff (at least among the 70 diners who completed both the in-restaurant and follow-up survey, representing a 56% response rate). By contrast, far fewer of the responsive diners’ memories were associated with leaving the restaurant. Ultimately, if you know what your customers are likely to remember, you are going to be in a better position to modify your food service offering, no matter whether you are operating a chain restaurant (like Pizza Hut) or a luxury hotel (see Ariffin et al., 2013). Knowing what is likely to stick in a guest’s memory, it becomes possible to imagine how one might want to design the experience differently (*cf.* Rowley, 2010; see also LaTour and Carbone, 2014, on the intriguing notion of ‘sticktion’). Note here also how it is likely that the guest’s memory of the experience (rather than the actual experience) that dictates their likelihood of booking a return visit and recommending the venue to others (i.e., through word of mouth; Walker, 2004; Khan et al., 2015).

One of my favorite examples of ‘consumer engineering’ (see Carbone and Haeckel, 1994) involves the use of scent to help enhance the guest’s memory of a high-end multisensory experience (Spence, 2017) by using scent after, and potentially also prior to, the guest’s trip (as at The Fat Duck restaurant in Bray; see Spence, 2017). For example, the guests at a number of Sensatori resorts are given a bottle of the resort’s signature scent (TUI Sensatori Scent) on departure (e.g., at their Rhodes resort opened in 2018^7^; see also Kilikita, 2018, for another example of scented memories of a tourist destination). By offering the guest the opportunity to take away the distinctive scent of the resort it will likely allow them to access more of the latter’s hopefully pleasurable memories, just like the Proustian moment (see Chu and Downes, 2000; Chu and Downes, 2002; Willander and Larsson, 2007). Bear in mind here only the fact that Zemke and Shoemaker (2007) found that a hotel’s signature scent had a considerable and positive effect on guests’ ability to recall their enjoyable hotel experiences. Meanwhile, in their book on the experience economy, Pine and Gilmore (1998, p. 58) mention the example of the Ritz-Carlton hotel in Naples, Florida where the decision was made to give away its 463 door knobs (engraved with the brand’s distinctive lion and crown insignia) to past guests as a physical reminder of a memorable stay when the hotel switched to an electronic door entry system using key cards. Once again, notice how sensory cues (tactile/visual) are being used to prime hopefully positive memories of a previous hotel experience.

## Public spaces in the hotel

While one might argue about the relative importance of the various different senses in triggering positive memories/recollections, the key point to note is that the senses interact and neither hoteliers nor their guests are aware of just how profoundly atmospheric sensory cues affect them, no matter whether it is the background music, the signature scent, or the ambient colour scheme that one is talking about (see also McGuire, 2016). Of course, beyond designing the multisensory guest experience to deliver a host of positive memories, there are also a number of functional benefits that may result from hoteliers’ attempts to engage their guest’s senses more effectively. We have already seen how aromatherapy scents can potentially be used to help guests relax (Pacelle, 1992; Spence, 2021c) while appetizing food scents may be (and have been) used to encourage the hotel’s guests to order more food and drink options (Hoppough, 2006; *cf.* Spence, 2022b).

An intriguing example of the recent foregrounding of fragrance in hotel design comes from the prominent use of the 540 Celsius fragrance in the recently-opened New York Baccarat Hotel (where rooms start at $1,500 a night; Thornhill, 2022).^8^ According to Hou (2021), such a prominent use of scent builds on the New York Gramercy Park Hotel’s use of the “Santal 33” scent back in 2015. The increasingly-common scenting of individual hotels, and the branded scenting of hotel chains, can be seen both as mirroring what has been going on in a range of other public spaces in recent decades (McGuire, 2017; see Spence, 2020a, for a review). Thompson Hotels spray their custom-designed perfume ‘Velvet’ in the guest rooms and open spaces (Wiedmann et al., 2016). In this case, rather than obviously delivering a functional benefit (in terms of relaxing the guests, say), the aim would appear to be simply to engage with another of the guest’s senses, by means of a deliberate targeting of the latter’s sensory ‘touch points’ (Lindstrom, 2005a,b; Krishna, 2010).

Luxury, safety, etc. are all multisensory concepts. As Jo Polmear, Creative Director of the Hunter Patel Creative Group notes: ‘Corridors in budget hotels will always be brightly lit to give guests reassurance and a feeling of safety. Whereas high-end hotels purposely create more moody, atmospheric areas to create an air of exclusivity and sumptuous luxury, as guests already feel assured by the heavy price-tag paid for their utmost security and wellbeing.’ (Thornhill, 2015). When it comes to conveying a sense of security in underground car parks (or public underground transport), choir music and bird song can both apparently be used to enhance perceived security, at least according to the results of an intriguing series of laboratory studies reported by Sayin et al. (2015).

Music obviously plays an important role in our experience of the built environment *–* think here only of the Muzak of decades gone by (Lanza, 2004; Prochnik, 2011). This is as true of the guest’s hotel experience (e.g., when entering the lobby) as it is elsewhere (e.g., in a shopping center or bar, say; Trompeta et al., 2022).^9^ The sound/music that greets customers in the lobby is very important to Ian Schrager, the Brooklyn-born entrepreneur responsible for fabled nightclub Studio 54 in New York. Schrager has been working with Marriott to launch *The EDITION* hotels in a number of major cities, including New York and London. Music plays a key role in the experience envisioned (?) by Schrager. As the entrepreneur put it in one interview: “The sound of a hotel lobby is often dictated by monotonous, vapid lounge muzak – a zombie-like drone of new jazz and polite house, with the sole purpose of whiling away the waiting time between check-in and check-out.” The music in the lobbies of The EDITION hotels is carefully-curated (Eriksen, 2014, p. 27). Another hotel that has become known for curating the sound for its guests is the Costes Hotel in Paris,^10^ (Di Donna, 2021).

At the same time, however, it is important to note how the thumping noise of the music from the nightclub/bar that is often also an integral part of the experience so often offered by these hip venues means that meticulous architectural design is required in order to limit the spread of unwanted noise throughout the rest of the building (e.g., so as not to disturb the sleep of those who may be resting in the rooms upstairs). There are some increasingly-sophisticated solutions – including the use of sound-absorbing panels, as well as active noise cancelation systems – to dampen unwanted sound in open spaces such as restaurants, offices, and hotels, however, the expense is sometimes deemed prohibitive (Clynes, 2012; Spence, 2021).^11^ That said, many hotels have undoubtedly found it rather more challenging to balance a thumping nightlife scene downstairs with the demands of guests upstairs who want a good night’s sleep (Sharkey, 2007).^12^

Even for more mainstream hotels, there is reason to consider the music playing in public spaces such as the restaurants and bars more carefully. This is both because it can provide a very effective means of helping to set the atmosphere, but also because it can be used to bias the customer’s decisions; i.e., when it comes to nudging their choices, e.g., concerning food/wine (e.g., see Areni and Kim, 1993; North et al., 1997, 1999; Zellner et al., 2017; and see Spence et al., 2019, for a review). Magnini and Thelen (2008) and Magnini and Parker (2009) have looked specifically at the role played by music in hotels and fine-dining restaurants. Ultimately, though, what is striking is how little awareness people normally have concerning the effect of the multisensory atmosphere on their own well-being, choices, and behavior (see also Gordon, 2009).

### Work and creativity: Welcome to the ‘hoffice’

It has long been noted that many business travelers use their room for work as well as relaxation (Levere, 2003). However, in recent years, there has been something of a change in role/function for many hotels, with an increasing interest in, expectation of, the provision of co-working space (in part linked to the recent Covid pandemic; see Accor, 2020). This is akin to the rise in the ‘coffice’ (Garside, 2004; Anon, 2014), in light of the increasing tendency for the coffee shop to become the sociable meeting place for today’s Millennial workers. Matched, in this case, by the growing interest from chains in the provision of co-working space. What, by analogy, I suppose might be christened the ‘Hoffice’ (i.e., ‘hotel’ plus ‘office’; which, I would suggest, sounds better than the other neoglism that I have come across - “workspitality”; Accor, 2020) At the same time, it is rare to come across a hotel meeting room that has been designed to foster creativity (Spence, 2021b; Robson, 2022). Too often, one finds oneself in a low-ceilinged windowless basement, with white walls, rectangular tables, and an absence of plants. Certainly, one of the least creative environments that one could imagine, and one that fails to harness any of the beneficial effects of the nature effect (Williams, 2017).

Those who were really wanting to deliver on the promise of offering spaces that foster creativity, or facilitate negotiation, should probably be aware of the literature on table shape and consensus building (Zhu and Argo, 2013), not to mention ceiling height and creative thinking (Meyers-Levy and Zhu, 2007; see Spence, 2017; Spence, 2021b). Furthermore, tips such as changing the ambient scent after a stressful meeting provides a simple sensory means (or ‘sensehack’; Spence, 2021b) to deliver an olfactory reset. The latest evidence concerning commensality and deal-making has implications for the business hotelier as well (see Spence, 2016, for a review). For instance, intriguing research from Balachandra (2013) reported in the *Harvard Business Review* attempted to quantify just how much of a potential benefit might accrue as a result of eating while negotiating a complex trade deal. Groups of MBA students (*N* = 132 in total) were set the task of finalizing the details of a complex joint venture agreement between two companies that had already been agreed in principle. In order to maximize the potential benefits for both sides, the negotiation required a degree of empathy and understanding for the other side’s position/needs, with the two sides needing to share information. Crucially, those deals negotiated by groups of students who had been fed would potentially have generated 6.7 million dollars more for the parties concerned. The students either ate in a restaurant or else were provided with their food in a conference room. A 12% increase in hypothetical profits was documented in the restaurant setting as compared to an 11% increase in the business conference room when food was brought in. Importantly, a control study with a further 45 MBA students demonstrated that it was sharing food, rather than completing some other task together, such as, for example, a jigsaw, that led to increased profitability.

## The guest bedroom

According to Ogle (2009), the hotel guestroom should be considered as the hotel’s core product. Ambient lighting (meaning table lamps and lamp shades and not just a single ceiling light) is often carefully introduced to create a feeling of calm and warmth, custom fabrics (stimulate both the eye, as well as the sense of touch, as the guest likely imagines how they would feel), and clever zoning can all help to make to improve the guest’s impression (Haslett, 2019; though see also Park et al., 2010).

### Sensory approaches to a good night’s sleep

One in three Brits said that they booked trips hoping to catch up on sleep (Delahaye, 2020). It is rather unfortunate, therefore, that research commissioned by Wyndham Hotels & Resorts suggests that 82% of Brits do not sleep any better in a hotel room than they normally would. Part of the reason for this is likely linked to the ‘First Night Effect’, which is likely to affect the majority of hotel guests (Agnew et al., 1966; Tamaki et al., 2016). The suggestion from the neuroscientists is that our brain stays alert whenever we stay somewhere new. In terms of possible sensory solutions to helping the guest sleep better, lavender has long been known to help promote sleep (Hardy et al., 1995; see also Kirk-Smith, 2003; Fismer and Pilkington, 2012). On a recent trip to New York, even the budget chain hotel I was staying at had a cart in the middle of the lobby at night for guests to take some lavender scent up to their room with them. That being said, according to the latest research, preferred scents may work best to help enhance self-reported sleep quality (Sabiniewicz et al., 2022). In one marketing-led intervention from a few years ago, the Travelodge chain (in the UK) trialed a range of scents in their bedsheets to relax guests. Some of the smells to be used were freshly cut grass, baby powder, apple pie and chocolate (see Press Release, 2008; Gordon, 2009). Bedroom air quality has also been shown to influence the quality of sleep (Strøm-Tejsen et al., 2016; see also Chan et al., 2009). Offering pillow menus also helps to target the sense of touch and has become increasingly popular.^13^ This can perhaps be thought of as a kind of tactile customization of the guest’s experience.

A recent survey of 2000 Travelodge Hotels in the UK reported that a soft shade of blue was the best colour in terms of helping their many guests to get a good night’s sleep (Best Bedroom Colors for Sleep, 2020). At the same time, of course, given how many problems revolve around noise, one might wonder whether the sound of a dishwasher would really help one get to sleep? A few years ago, easyHotel launched a lullaby service for guests to make them feel more at home, available by Spotify app (Whitelocks, 2018). The options included a constant traffic hum (but without the honking horns), or perhaps the sound of rainfall and lapping waves. In fact the full track listing, as reported in the press was: (1) Washing machine in the distance; (2) Dishwasher in the distance; (3) Light hoovering downstairs; (4) Free-moving traffic (no horns); (5) Cat purring; (6) Clothes drier; (7) Electric wall clock; (8) Distant calm conversation; (9) Radiator pipes; (10) The sound of a shower; (11) Dog quietly barking; and (12) Gardening program. While originally available at just three locations (Glasgow, Newcastle, and Croydon), the suggestion was that this would be rolled out across the network of hotels.^14^ Meanwhile, the Point Hotel Barbaros in Istanbul has put several measures in place to try and help guests sleep, including special mattresses and pillow menus, as well as books to promote sleep by beds, and a lullabies soundtrack (Vardarı and Arapı, 2017), thus offering a multisensory approach to this perennial guest complaint.

The Peninsula Beverly Hills offer personalized room cents to guests in their room (Vardarı and Arapı, 2017), inspired by the locale. In particular, their new signature bathroom amenities, launched in January 2021, are created with the aim of reflecting the location of each of the group’s ten different properties around the world. The idea is to give the guest an immersive scent-sory experience through each individual bespoke fragrance that is used to scent the bathroom amenities. According to a spokesperson for the brand, the aim is “to encourage a distinctive sensory immersion in each of our hotel locations.” (see Villa-Clarke, 2020). It is interesting to contrast this local, bespoke approach to scent the bathroom amenities with the ubiquitous use of the same branded scent at all consumer touch points by the likes of Singapore Airlines, where the staff, hot towels, all smell of Stefan Floridian Water (see Spence, 2021a).

The local theming also applies at the MGM Grand Hotel & Casino in Las Vegas, according to Gilmore and Pine II. (2002a), p. 88. The guests who stay there may choose to be woken-up by the sound of recorded voices of celebrities who have performed there previously. The guests are likely to be awakened by a different star each time they visit. There is, then, often a choice between a local flavor to the sensory intervention, and a consistent branded signature sensation, be it a scent, a jingle, a particular colour scheme etc.

### Temperature

An inverse correlation has been documented between price and temperature in retail stores in North America (with temperatures ranging between 68–78°F; see Salkin, 2005, ‘Shivering for luxury’). That is, the higher the price point, the lower the store temperature. According to certain researchers, this may influence people’s thinking styles, potentially nudging them toward more rational/emotional purchases (see Park and Hadi, 2020). Is the same true of hotel/resort accommodation, one might well ask? Well, this would seem unlikely if, as Heschong (1979, p. 44) has suggested, the association between luxury and store temperature actually originated from expensive stores in North America being the first to be able to afford air-conditioning (thus establishing the inverse correlation between ambient store temperature and price). As such, there is no particular reason to believe that the same relationship will necessarily have been internalized in the hospitality setting.

Nevertheless, it still makes sense for the hotel manager to pay careful attention to temperature/thermal comfort and make an informed choice about how to meet one’s guests’ needs. Interestingly here, when researchers assessed the indoor climate that people chose for their own homes, regardless of where in The States people lived (*N* = 37; e.g., all the way from Alaska to Florida), the settings in terms of temperature (*M* = 27.3°C) and humidity (*M* = 16.15 hPa) most closely matched the west central Kenya where humanity emerged several million years ago (Just et al., 2019). According to my Oxford University colleague, the sleep expert Russell Foster suggested that keeping the temperature at 18–22°C should help guests to sleep better (Delahaye, 2020). At the same time, however, one also needs to consider individual differences and, in particular, gender differences in thermal comfort. Indoor temperature specifications from building regulations are, perhaps unsurprisingly, based on the comfort of a middle-aged white man. But the evidence suggests that women prefer, and work better, when the ambient temperature is several degrees higher (see Spence, 2021b). According to the results of one study, European and North American men actually prefer an ambient temperature that is an average of 3.1°C lower than the 25.2°C that is preferred by Japanese women. Due to men having more heat-producing muscle mass than women, meaning that their metabolic rates are as much as 30% faster (see Kingma and van Marken Lichtenbelt, 2015; Chang and Kajackaite, 2019). Nevertheless, given such sex differences in thermal comfort, it is clearly going to be a challenge to please everyone all of the time. However, given how frequently problems with the temperature rank among the top concerns/complaints of hotel guests, it surely has to make sense to store information about a guest’s preferences, or simply based on their stated sex, and adjust the temperature of their room on arrival. One could, I suppose, think of this as thermal personalization/customization (*cf.* Suh et al., 2015). Once again, though, taking one’s guests’ thermal comfort seriously requires moving beyond a visually-dominant mind-set in hospitality.

## Hospitality: Fine dining and drinking in hotels

There has long been interest in how to optimize the multisensory environment in the context of the hotel restaurant (Lampi, 1973). According to research reported by Lundberg (1994), guests typically spend two hours a day (while awake) in their guestroom, and a further two hours at various on-site facilities such as hotels food and beverage (F&B) outlets. There has also been a great deal of interest from hotel owners in trying to ensure that their food offerings are profitable (TMI Spy Hotels and Leisure, 2015), something that has become especially challenging in the current climate where, in many cities, guests can nowadays order in the food they want *via* food delivery apps such as Deliveroo and Just Eat.

It is certainly striking to see how a number of high-end experiential drinking and dining concepts have been based in hotels in recent years (Wu and Liang, 2009; Galeano Balaguera, 2022; Martínez Polo, 2022). Here, one might think of everything from London’s Berkeley Hotel *Out of the Blue* multisensory cocktail experience (Ellis, 2017; now closed) through to the rather more successful *Sublimotion* at the Hard Rock Hotel in Ibiza. According to press reports, the latter venue is the world’s most expensive dining experience (Driver, 2014). According to one press report concerning the latter venue: “Michelin-starred chef Paco Roncero is behind the five-star resort’s exclusive concept, which aims to take 12 guests at a time ‘an experience for all the senses’.” (Driver, 2014).

Those wanting to see what the future may hold in terms of restaurant design should take a look at the Goji Kitchen & Bar at the Marriott Bund hotel in Shanghai. The decor in this futuristic dining space actually changes to give the restaurant one of two distinct feels, depending on the time of day.^15^ This is undoubtedly a very expensive solution, but it is also an acknowledgement that decor and atmosphere really do matter (Spence, 2020d). It stands as testament to the importance of the atmospheric component of ‘the everything else’ (i.e., context and atmosphere) to mealtimes (see also Heung and Gu, 2012). Relevant here, one need only think of the global success of the Irish Pub concept in recent decades to understand just how important getting the atmosphere right can be (Bloodworth, 2017). Postrel (2004, p. 20) makes a similar point when she notes that: “Starbucks is a prime example of an aestheticaly-designed brand sensorium: they do not just serve coffee, but create an environment that attends to rich textures, colors, aromas, taste treats and music, and so induces a respite from the hectic world. Starbucks, she writes, ‘is to the age of sensory aesthetics what McDonalds was to the age of convenience or Ford was to the age of mass production.’”

The Adam & Eve Hotel^16^ has The Blind Restaurant (serving up to 16 people) adopting the dine-in-the-dark concept in its Antalya Belek resort (as but one of the multiple dining options on offer; Vardarı and Arapı, 2017). Although it is difficult to find anyone who says that the food tastes especially good in such restaurants (e.g., see Spence and Piqueras-Fiszman, 2012; Spence and Piqueras-Fiszman, 2014), this nevertheless clearly fits in as experiential dining, and by eliminating light provides another means of changing the atmosphere for guests. In terms of destination tourism resorts, it is worth noting that more tourists than ever before now choose their holiday destination based on the cuisine that they expect to find there (Amey, 2015; see also Cohen and Avieli, 2004; Edensor, 2007; Berbel-Pineda et al., 2019).

The Tui Sensatori resort in Rhodes also trialed a multisensory experiential dining concept as an optional extra for guests at the all-inclusive venue (see Tui, 2018). Meanwhile, at Sofitel’s The Grand hotel in Amsterdam’s Restaurant, The Bridge’s, stimulating the senses of guests from around world (especially the US, UK, Germany and Middle-East), with *The Grand Carousel of Senses* (Derval, 2010, pp. 59–61; Matthewson, 2009). This part of a festival Stars, Food & Art, gathering 12 chefs with 29 Michelin stars. Music composed to accompany each meal, complemented and amplified by scents by DJ Fragrances. The plan was to reproduce this sensory festival at the Softitel’s hotels in other cities such as Munich, Rabat, New York, and Abu Dhabi. There has been an increasing focus on fine dining in the luxury end of the hotel sector (e.g., see the Six Senses Group and Sensatori resorts), in part because of the opportunity such encounters provide to engage with the guest’s more emotional senses (Wiedmann et al., 2016), and also to hopefully deliver memorable, pleasurable, multisensory experiences.

## Biophilic design

There has also been an increasing focus on wellness by hoteliers in recent years (Wiedmann et al., 2016; Kilikita, 2018). At the same time, there has also been an explosive growth of interest in the emotional and cognitive benefits associated with exposure to nature. While biophilic design is often described in terms of reproducing the greens and blues of nature and water (e.g., Nichols, 2014; Williams, 2017), the benefits of listening to, smelling, an even feeling nature should not be underestimated either (see Spence, 2021a; Khozaei et al., 2022). A sign of things to come is represented by the Joali Being resort/retreat in the Maldives. There, biophilic design principles have been integrated into an architecturally striking setting offering transformational wellbeing programs (Hill, 2021). Intriguingly, given the theme of this review, guests are also offered the opportunity to enter a sensory deprivation room. Biophilic design is undoubtedly on the rise, both in hospitality settings as well as elsewhere. What is more, the guests can be connected to nature *via* any of their senses. Although more research is needed, it is also likely that congruency and multisensory stimulation should help to enhance the benefits of the nature effect too (Spence, 2021b). Linking back to a theme that was discussed earlier, there may also be a connection between indoor greenery and creativity (Shibata and Suzuki, 2004). Multisensory experience design (especially when it is related to the benefits of biophilic design) is likely to play an important role in the design of medical hotels, a sector that has grown substantially in recent years (Han and Hyun, 2014). However, reviewing the rapidly expanding literature on biophilic design is beyond the scope of this review. If anything, though, the evidence currently suggests that exposure to actual nature may sometimes be more restorative than a digital rendition (see Nabham and St. Antoine, 1993; Kjellgren and Buhrkall, 2010; though see also Krieger, 1973), though oftentimes either version would appear to be beneficial.

## Personalizing/customizing the experience

Given the contemporary importance of being able to personalize, or customize, the design of experiences, one important question concerns how hoteliers can personalize their guests’ multisensory experiences. This is something guests increasing appear to want, at least in the context of luxury hotels (Shahid and Paul, 2022). As in high-end fine-dining, there is the opportunity to either let your guests know that you have been googling them, or not (see Spiegel, 2014; Spence, 2017). Loyalty card programs potentially provide an excellent means of acquiring useful information with which to customize/personalize a guest’s stay (assuming that the hotel has access to that information). So, for example, making sure that guests are offered the same room they stayed in before will likely help to reduce the first night effect, and hence enhance the quality of the sleep they get, at least on the first night (see above).

Relevant here, a number of the airlines have been offering their customers different aromatherapy solutions at different times, to help enhance passenger comfort (Garcia, 2016). Just take Virgin Atlantic Upper Class which, according to Garcia, offers: “Passengers in the airline’s Upper Class receive a hot towel infused with de Mamiel essential oils, which vary according to time of day. During daytime flights, an aromatic blend of bergamot, sweet orange and yang ylang, called Enliven, helps passengers relax and feel refreshed; during overnight flights, the fragrance is called High Altitude and includes fragonia, eucalyptus and lavender to help boost immunity and induce sleep. Meanwhile, Emirates offers its first-class passengers Matakana Botanicals’ Sleep and Focus Sniff Boxes (concentrated aroma pearls), that are designed to help the passengers on board relax and to counter the effects of jet lag. Note here that it helps if these kinds of sensory experiences can be built into rituals. So, for example, at Six Senses Hotels Resorts Spas they stage rituals for their guests to participate in. Beyond simply delivering memorable scents and relaxing sounds, rituals are an especially effective ploy for hooking and reeling in the consumer (Malefyt, 2014).

According to a survey conducted in 2000 by Embassy Suites Hotels, travelers can be segmented into two personality types: ‘Upstairs’ and ‘Downstairs’ guests. The former tend to be more introverted and spend less time in public areas, preferring private spaces such as bedrooms, over more social spaces such as lobbies and lounges. Unsurprisingly, ‘Downstairs’ personalities are more extroverted and tend to spend more time in public areas (see Ogle, 2009, p. 161). Here there may be scope to combine the notion of personality with Wober’s (1966), Wober’s (1991) intriguing notion that there may be different ‘sensotypes’, that groups of people exhibiting different kinds of sensory engagement. Segmenting one’s customer base into different sensotypes might then allow one to customize or even personalize the multisensory offering, be it in terms of thermal comfort, noise, scent, etc. One London hotel has apparently even gone so far as to over its own ‘Five Senses Concierge’ on arrival (Lee et al., 2019). Such an approach undoubtedly helps to draw attention to a hotel’s sensory credentials. Looking to the future, the emergence of the Internet of Things (IoT) in upscale hotels may help to further curate a guest’s multisensory experiences (Pelet et al., 2021). According to the latter researchers, based on interviews with hotel managers, and a confirmatory online study with 357 hotel guests, the senses of smell, hearing and sight affected guests’ emotions, while the senses of touch, hearing and sight impacted guests’ affective experiences. Meanwhile, the senses of smell and taste influenced guests’ well-being (what the authors refer to as ‘eudaimonism’).

## Conceptual models of the relative importance of the senses in multisensory marketing

Wiedmann et al. (2018) developed a conceptual model of multisensory marketing in the luxury hotel sector that also incorporated brand experience, customer perceived value, and brand strength. The conceptual framework operationally captures the different constructs as well as highlighting the assumptions regarding the possible causal relationships amongst them based on the available knowledge. Participants were asked to rate their agreement with a range of statements on a 5-point scale from ‘strongly disagree’ to ‘strongly agree’. According to Wiedmann and colleagues’ evaluation of a qualitative online survey of 552 German consumers who were familiar with luxury hotels, all of the sensory drivers were significant, with visual and gustatory perception suggested to be the most powerful. Acoustic and olfactory drivers each played a significant but slightly less important role, while haptic perception played the least important role. According to the researchers involved, their analysis of the questionnaire responses using structural equation modeling supported the majority of the 15 hypotheses concerning the relation between variables captured within their conceptual model.

However, a closer look at the multisensory component of the questionnaire raises a number of concerns with regard to the way in which at least certain of the data was coded. In particular, in order to probe multisensory marketing, the respondents were asked to rate their level of agreement with 4–7 statements for each of five senses. So, for example, under the Gustatory header we find statements such as ‘The meals in luxury hotels are a real pleasure.’ However, given that 75–95% of what we think we taste, we actually smell (i.e., *via* retronasal olfaction; see Spence, 2015a), the majority of the questions that the researchers designed to assess gustation, should perhaps better be considered as targeting smell, flavor, or at the very least a mixture of gustation and olfaction. Let us take a closer look at another of the questions in the Gustation section of Wiedmann and colleagues’ questionnaire, namely ‘My mouth is watering by looking at the menu in luxury hotels’, one might legitimately wonder whether this should not be in the vision section. After all, there has been a huge growth of interest in food porn/gastroporn, and while the subject matter may well stimulate the chemical senses, the pleasure is fundamentally linked to the visual sense, not gustation (Spence et al., 2016). Added to these problems, take a third question in the gustatory section, namely ‘The beverages in luxury hotels are very delicate’. Now maybe this is a translation issue, but it is not obvious (to your author) that a drink being ‘delicate’ is necessarily a particularly important/relevant attribute as far as the food offering is concerned.

A somewhat different hierarchy of sensory cues was reported by Suh et al. (2015). The latter researchers studied the physical environmental factors contributing to guest loyalty in 5-star metropolitan hotels in Korea. They analyzed questionnaire responses from 422 guests who were waiting to check-out in the lobby (comprising 42% leisure travelers, 30% business, and 10% meeting/conference, and the 18% did not respond). They concluded “that marketers should pay more attention to managing temperature within the property. Further, the current study identified that music has the most significance in affecting satisfaction, followed by odor/aroma, noise/sound level, and air quality. Thus, in order to increase customer satisfaction, it will be critical that hotels play pleasant background music within their common areas.” (Suh et al., 2015, p. 745; see also Walls, 2013, on the importance of ensuring that the temperature is as pleasant as possible). Given such a discrepancy in the sensory hierarchy of importance, further research is therefore clearly needed in order to better understand whether such differences reflect cultural variation in terms of hotel guests’ expectations in different countries, or whether instead they may point to there being a different hierarchy of sensory importance in luxury as compared to other sectors of the hotel market.

Given that some events stick in memory better/longer than others (e.g., LaTour and Carbone, 2014), it would be interesting in future research to track how such responses to questions targeted at assessing the relative importance of the different senses change over time by administering such questionnaires at different points in time around a guest’s stay in a particular type of hotel. Should the results of such a longitudinal study throw up any changes in the pattern of sensory dominance over time (*cf.* Schifferstein, 2006), then this might also provide another explanation for why the two studies just mentioned should have revealed different hierarchies of the senses.

What is more, one other important point to note is how our perception in one sense is very often influenced by the stimuli presented simultaneously in another (Spence, 2021b). Often such multisensory and crossmodal effects occur without awareness and are automatic. Hence, it is difficult to know exactly how important such cross-sensory factors are when the hotel customer says, for example, that the music is too loud, or the temperature too warm. It may be that these responses could be mitigated simply by turning down the lights or changing the lighting colour (Spence, 2020b), rather than necessarily addressing the sense that the complaints seem to reference.

## Conclusion

Wiedmann et al. (2016) conclude their article on luxury by suggesting that: “luxury hotels have to create superior experiences and memorable holidays. In a luxury context, sensory stimulation is not a hunt for ‘the next big thing’, but rather a concentration on the subtle refinement of colors, tastes, fragrances, sounds and textures in an orchestrated holistic concept. Especially in an age of information overload and sensory overstimulation (Malhotra, 1984), a true luxurious experience provides a calming relaxation – a perfect example is the global trend of ‘tech-free zones’ and ‘digital detox’ holidays.” This can, I would argue, all be seen from the perspective of ‘sensehacking’ multisensory hotel design (Spence, 2021b). However, while there is undoubtedly growing interest in applying ideas related to sensory marketing in hotel design (e.g., Amorntatkul and Pahome, 2011; Vardarı and Arapı, 2017; Kim et al., 2020; Shehata and Alaswadi, 2022; see also Ali and Ahmed, 2019), it is striking how there would appear to be far less published research extending ideas related to sensory marketing (Hilton, 2015; Wörfel et al., 2022), experiential marketing, sensory branding (Zha et al., 2022), and the experience economy, to the highly-profitable domain of hotels, when compared to the extensive discussion of these topics in the context of retail (e.g., see Shahid et al., 2022; Wörfel et al., 2022).

Looking to the future, techniques such as Emerging Pattern Mining may provide a useful means of identifying emerging hotel preferences (Li et al., 2015). At the same time, there have been a number of examples of destination marketing using virtual reality (VR) in order to try and tempt people to book a trip (Adams, 2016). However, one of the challenges with the use of such emerging technologies in a multisensory context is that they can typically only effectively stimulate a user’s visual and auditory senses.

Key insights to emerge from this review include recognizing the importance of effectively stimulating the guest’s non-visual senses; Recognizing the ubiquitous multisensory/crossmodal influences on a hotel guest’s perception; Recognizing the role of biophilia/the nature effect (e.g., Williams, 2017; Spence, 2021) in their guests’ sense of well-being; Considering how to curate/personalize the guest’s multisensory experience; Recognizing that a guest’s memory of a hotel experience is likely to be importantly different from their experience in the moment. Those interested in probing the hotel guest’s multisensory experience need to be aware of such temporal factors, and should also be thinking carefully about how to engage their guest’s senses prior to arrival and long after they have left. Of course, beyond the important role played by sensory factors, there are likely also to be a number of important cognitive and affective factors influencing a guest’s choices as well (Kim and Perdue, 2013).

There is also growing interest in designing scentscape, scent-mapping, or scent-sory journeys. Notice, though, how the latter approach may require the selection of multiple scents. Sensatori multisensory pods in the lobby of the Tui Sensatori resort in Rhodes (opened 2018), as designed by Condiment Junkie,^17^ (Gray, 2019), provide an interesting example on which to end here. Guests can simply climb into one of the three pods, which deliver scent, touch, sound, and video (one might even consider whether they engage proprioception/bodily senses given that the guests need to climb into the pods in the first place).^18^ These multisensory oases/cocoons re designed to help transport the guests away from any stresses associated with their travel to the resort and involve several multisensory biophilic elements. There is a growing recognition that travelers are attracted to such immersive installations and multisensory experiences (e.g., see Eskins, 2022; Stead et al., 2022). Ultimately, therefore offering a memorable luxury hotel experience increasingly involves providing the right diet of multisensory stimulation/immersion to guests (see Kilikita, 2018; Wiedmann et al., 2018; Gray, 2019; Lee et al., 2019; Di Donna, 2021; Shahid and Paul, 2022).

## Author contributions

The author confirms being the sole contributor of this work and has approved it for publication.

## Conflict of interest

The author declares that the research was conducted in the absence of any commercial or financial relationships that could be construed as a potential conflict of interest.

## Publisher’s note

All claims expressed in this article are solely those of the authors and do not necessarily represent those of their affiliated organizations, or those of the publisher, the editors and the reviewers. Any product that may be evaluated in this article, or claim that may be made by its manufacturer, is not guaranteed or endorsed by the publisher.

## Footnotes

^1^The latter quote seemingly copied directly from the hotel advertisement itself.

^2^There is a great short video created by the Future of Storytelling (FoST) Institute in New York in 2016, should you wish to find out more about the rules of multisensory perception: https://futureofstorytelling.org/video/charles-spence-sensploration.

^3^As one commentator put it: "We’re all preoccupied with what stimulates us visually or audibly, and I think smell gets short shrift. Why not add it to the design mix?" (Gaillard, quoted in Burr, 2008, p. 106).

^4^ https://www.adamevehotel.com/

^5^A lack of cleanliness, remember, one of hotel guests’ top complaints, according to one of the surveys mentioned earlier.

^6^In fact, according to Masters (2022), the Hilton-owned DoubleTree hotel chain gives out 25 million chocolate-chip cookies to guests every year. Focus group research reveals that the cookie has real ‘sticktion’ – it’s something guests really remember (Masters, 2022).

^7^ https://www.tui.co.uk/press/luxury-holiday-concept-tui-sensatori-celebrates-10-years-sense-fuelled-holidays-new-rhodes-resort-gets-ready-opening/

^8^The scent apparently smells something like a cross between ‘burnt jam’ and ‘money’ or ‘smoke’ and ‘burnt candy’ (see Hou, 2021).

^9^Here, one might also consider the Abercrombie & Fitch clothing brand. For a number of years, the chain managed to craft a distinctive dance sound to match the dark nightclub-like appearance of their interiors (though see Elliott, 2014).

^10^ https://www.hotelcostes.com/en/music

^11^Back in 1935, it was the sound of a seal that kept one of the guests staying at the Pierre Hotel in New York awake. The offending animal was eventually tracked down to the Central Park Zoo, and moved to the Brooklyn Zoo to deal with the problem (see Prochnik, 2011, p. 201). It is perhaps hard to imagine the hoteliers of today dealing with complaints in such an efficient and conscientious manner.

^12^Or on the rooftop at the Standard Highline in The Meatpacking District in New York City. I have been kept awake all night by the nightclub on the roof for both of my visits to the venue (see https://www.standardhotels.com/new-york/properties/high-line), which undoubtedly has stunning views (see Howes, 2014, on the visual dominance in the experience of high-rise).

^13^ https://www.tui.co.uk/destinations/europe/greece/crete/lyttos-beach/hotels/tui-sensatori-atlantica-caldera-palace.html

^14^One of the problems with many such sensory marketing interventions is that they tend to be launched in order to secure press coverage and, of course, the wackier the proposed solution, the more newsworthy it is, and then shortly thereafter the intervention is quietly canceled/dropped. Cynically, my take is that such marketing-led interventions are really targeted at securing ‘free’ marketing for the brand and not actually about improving guest well-being at all.

^15^See the video at https://travel-brilliantly.marriott.com/our-innovations/goji-kitchen-and-bar. As Matthew von Ertfelda, VP of Innovation for Marriott, says in the video: ‘We will not only feed your appetite but we will ignite your senses.’

^16^ https://www.adamevehotel.com/

^17^ https://www.tuigroup.com/en-en/media/stories/2018/2018-02-22-tui-sensatori-celebrates-anniversary

^18^According to US marketing professor, Aradhna Krishna Six Senses Hotels Resorts Spas may be said to "own" the senses the same way Apple came to "own" touch when it released the iPod Touch. The senses are not only given in the name of the Six Senses, they also pervade the publicity and presumably the experience of these resorts (e.g., https://www.sixsenses.com/en). At the same time, however, it may be worth considering whether all the sensory language and of sensory marketing talk necessarily always lives up to the claims that are made for it (see Chapter 5: How capitalism came to its senses – and yours: the invention of sensory marketing in Howes and Classen, 2014).

## References

[ref2] Accor (2020). Co-working: hotels are re-inventing themselves. *Hospitality Net*. September 17. Available at: https://www.hospitalitynet.org/news/4100694.html (Accessed November 27, 2022).

[ref3] Adams R. L. (2016). Five ways virtual reality will change the world. *Forbes*. October 17. Available at: https://www.forbes.com/sites/robertadams/2016/10/17/5-ways-virtual-reality-will-change-the-world/#5795b7312b01 (Accessed November 27, 2022).

[ref4] Agnew H. W. Jr. Webb W. B. Williams R. L. (1966). The first night effect: an EEG study of sleep. Psychophysiology 2, 263–266. doi: 10.1111/j.1469-8986.1966.tb02650.x, PMID: 5903579

[ref5] Ali E.-H. M. Ahmed M. O. (2019). Sensory marketing and its effect on hotel market-share: perception of hotel customers. J. Hosp. Tour. Manag. 7, 116–126. doi: 10.15640/jthm.v7n1a12

[ref6] Amey K. (2015). Food for thought! One-fifth of Brits admit that local cuisine is the most important factor when choosing a holiday destination. Daily Mail Online, August 4. Available at: https://www.dailymail.co.uk/travel/travel_news/article-3184743/Food-thought-One-fifth-Brits-admit-local-cuisine-important-factor-choosing-holiday-destination.html (Accessed November 27, 2022).

[ref7] Amorntatkul N. Pahome T. (2011). How sensory marketing applies to the hotel and restaurant industry in Thailand. [Master Thesis, Mälardalen University]. Available at: http://tourismlibrary.tat.or.th/medias/MAL0101/MAL0101_abstract.pdf (Accessed November 27, 2022).

[ref8] Anon. (2014). The coffice: the future of work? *The Guardian*, January 5. Available at: https://www.theguardian.com/money/shortcuts/2014/jan/05/coffice-future-of-work (Accessed November 27, 2022).

[ref9] Areni C. S. Kim D. (1993). The influence of background music on shopping behavior: classical versus top-forty music in a wine store. Adv. Consum. Res. 20, 336–340.

[ref10] Ariffin A. A. M. Nameghi E. N. Zakaria N. I. (2013). The effect of hospitableness and servicescape on guest satisfaction in the hotel industry. Canadian J. Adm. Sci. 30, 127–137. doi: 10.1002/cjas.1246

[ref11] Aroche D. (2015). Never heard of Sensploration? Time to study up on epicure’s biggest luxury trend. *LuxeEpicure*, December 22. Available at: http://www.justluxe.com/lifestyle/dining/feature-1962122.php (Accessed November 27, 2022).

[ref12] Balachandra L. (2013). Should you eat while you negotiate? *Harv. Bus. Rev.*, January 29. Available at: https://hbr.org/2013/01/should-you-eat-while-you-negot/ (Accessed November 27, 2022).

[ref13] Bentley P. R. Fisher J. C. Dallimer M. Fish R. D. Austen G. E. Irvine K. N.. (2022). Nature, smells, and human wellbeing. Ambio 52, 1–14. doi: 10.1007/s13280-022-01760-w, PMID: 35849312 PMC9289359

[ref14] Berbel-Pineda J. M. Palacios-Florencio B. Ramírez-Hurtado J. M. Santos-Roldán L. (2019). Gastronomic experience as a factor of motivation in the tourist movements. Int. J. Gastron. Food Sci. 18:100171. doi: 10.1016/j.ijgfs.2019.100171

[ref1] Best Bedroom Colors for Sleep. (2020). Available at: https://oursleepguide.com/best-bedroom-colors-for-sleep/ (Accessed November 27, 2022).

[ref15] Bloodworth A. (2017). Why Irish pubs became the biggest food and drink export since McDonald’s. *Vice (Munchies)*, August 22. Available at: https://www.vice.com/en/article/a3d8gb/why-irish-pubs-became-the-biggest-food-and-drink-export-since-mcdonalds (Accessed November 27, 2022).

[ref16] Bowles N. (2019). How to feel nothing now, in order to feel more later. *The New York Times*, November 7. Available at: https://www.nytimes.com/2019/11/07/style/dopamine-fasting.html (Accessed November 27, 2022).

[ref17] Bravo-Moncayo L. Reinoso-Carvalho F. Velasco C. (2020). The effects of noise control in coffee tasting experiences. Food Qual. Prefer. 86:104020. doi: 10.1016/j.foodqual.2020.104020

[ref18] Bridge M. (2018). Cut your stress level by taking a Facebook holiday. *The Times*, April 6, 13.

[ref19] Bridge M. Knowles T. (2019). Tech titans snub friends to avoid sensation overload. *The Times*, November 19, 27.

[ref20] Burr C. (2008). The sweet smell of the modern hotel. *T+L Journal*, June, 104–106.

[ref21] Carbone L. P. (2004). Clued in: How to Keep Customers Coming Back Again and Again. Upper Saddle River: Prentice Hall.

[ref22] Carbone L. P. Haeckel S. H. (1994). Engineering customer experiences. Mark. Manag. 3, 8–19.

[ref23] Chan W. Lee S. Chen Y. Mak B. Wong K. Chan C.. (2009). Indoor air quality in new hotels’ guest rooms of the major world factory region. Int. J. Hosp. Manag. 28, 26–32. doi: 10.1016/j.ijhm.2008.03.004

[ref24] Chang T. Y. Kajackaite A. (2019). Battle for the thermostat: gender and the effect of temperature on cognitive performance. PLoS One 14:e0216362. doi: 10.1371/journal.pone.0216362, PMID: 31116745 PMC6530830

[ref25] Chu S. Downes J. J. (2000). Odour-evoked autobiographical memories: psychological investigations of Proustian phenomena. Chem. Senses 25, 111–116. doi: 10.1093/chemse/25.1.111, PMID: 10668001

[ref26] Chu S. Downes J. J. (2002). Proust nose best: odours are better cues of autobiographical memory. Mem. Cogn. 30, 511–518. doi: 10.3758/BF03194952, PMID: 12184552

[ref27] Clynes T. (2012). A restaurant with adjustable acoustics. *Popular Science*. Available at: http://www.popsci.com/technology/article/2012-08/restaurant-adjustable-acoustics (Accessed November 27, 2022).

[ref28] Cohen E. Avieli N. (2004). Food in tourism: attraction and impediment. Ann. Tour. Res. 31, 755–778. doi: 10.1016/j.annals.2004.02.003

[ref29] Cotter K. N. Silvia P. J. Bertamini M. Palumbo L. Vartanian O. (2017). Curve appeal: exploring individual differences in preference for curved versus angular objects. i-Perception. 8, 1–17. doi: 10.1177/204166951769302 PMC5405906 28491269

[ref30] Crouse T. (2010). The Influence of Ambient Scent on Hotel Guests’ Responses (Publication Number 979494). Concordia University, Montreal, QC.

[ref31] Dazkir S. S. Read M. A. (2012). Furniture forms and their influence on our emotional responses toward interior environments. Environ. Behav. 44, 722–732. doi: 10.1177/0013916511402063

[ref32] De Lange M. Debets L. Ruitenburg K. Holland R. (2012). Making less of a mess: scent exposure as a tool for behavioral change. Soc. Influ. 7, 90–97. doi: 10.1080/15534510.2012.659509

[ref33] de Vries J. (1997). The Five Senses: If You Lose These Senses You Lose Your Sense of Living. Edinburgh, Scotland: Mainstream Publishing Company.

[ref34] Delahaye J. (2020). Sleep expert shares top tips for getting a decent night’s rest in a hotel room. *The Mirror*, March 13. Available at: https://www.mirror.co.uk/travel/news/sleep-expert-shares-top-tips-21686008 (Accessed November 27, 2022).

[ref35] Derval D. (2010). The Right Sensory Mix: Targeting Consumer Product Development Scientifically. Heidelberg: Springer.

[ref36] Di Donna J. (2021). Hospitality is the kingdom of sensory marketing. *Hospitalitynet*, April 16. Available at: https://www.hospitalitynet.org/opinion/4103968.html (Accessed November 27, 2022).

[ref37] Driver C. (2014). Don’t forget to tip! New hard rock Hotel in Ibiza to open the most expensive restaurant in the world – at £1,235 a HEAD. *Daily Mail Online*, April 21. Available at: http://www.dailymail.co.uk/travel/article-2609619/New-Hard-Rock-Hotel-Ibiza-open-Sublimotion-expensive-restaurant-world.html (Accessed November 27, 2022).

[ref38] Edensor T. (2007). “Sensing tourism spaces,” in Travels in Paradox. eds. Minca C. Oakes T. (London: Rowman & Littlefield), 23–46.

[ref39] Elliott A. F. (2014). Lights up, sound down, clothes on: Abercrombie & Fitch tones down its nightclub-themed stores in a bid to win back disinterested teens. *Daily Mail Online*, May 23. Available at: https://www.dailymail.co.uk/femail/article-2637492/Lights-sound-clothes-Abercrombie-Fitch-tones-nightclub-themed-stores-bid-win-disinterested-teens.html (Accessed November 27, 2022).

[ref40] Ellis D. (2017). Out of the blue: Berkeley hotel launch secret cocktail menu promising to change the way we drink. *The Evening Standard*, November 14. Available at: https://www.standard.co.uk/go/london/bars/out-of-the-blue-berkeley-hotel-launch-secret-cocktail-menu-promising-to-change-the-way-we-drink-a3690976.html (Accessed November 27, 2022).

[ref41] Eriksen L. (2014). Room with a cue. B&O Play 3, 26–27.

[ref42] Errajaa K. Legoherel P. Daucé B. (2018). Immersion and emotional reactions to the ambiance of a multiservice space: the role of perceived congruence between odor and brand image. J. Retail. Consum. Serv. 40, 100–108. doi: 10.1016/j.jretconser.2017.08.016

[ref43] Eskins J. (2022). How immersive installations are attracting travellers. *The Globe and Mail*, February 1. Available at: https://www.theglobeandmail.com/life/travel/article-how-immersive-installations-are-attracting-travellers/ (Accessed November 27, 2022).

[ref44] Fenko A. Loock C. (2014). The influence of ambient scent and music on patients’ anxiety in a waiting room of a plastic surgeon. HERD 7, 38–59. doi: 10.1177/193758671400700304, PMID: 24782235

[ref45] Ferrari R. (2015). Writing narrative style literature reviews. Med. Writ. 24, 230–235. doi: 10.1179/2047480615Z.000000000329

[ref46] Field T. (2001). Touch. Cambridge: MIT Press.

[ref47] Fismer K. L. Pilkington K. (2012). Lavender and sleep: a systematic review of the evidence. Europ. J. Integr. Med. 4, e436–e447. doi: 10.1016/j.eujim.2012.08.001

[ref48] Furley P. Goldschmied N. (2021). Systematic vs. narrative reviews in sport and exercise psychology: is either approach superior to the other? Front. Psychol. 12:685082. doi: 10.3389/fpsyg.2021.685082, PMID: 34305741 PMC8299000

[ref49] Galeano Balaguera P. (2022). Comida sensorial, la nueva apuesta de los hotels. *Portafolio*, July 15. Available at: https://www.portafolio.co/tendencias/comida-sensorial-la-nueva-apuesta-de-los-hoteles-568247 (Accessed November 27, 2022).

[ref50] Gallace A. Ngo M. K. Sulaitis J. Spence C. (2012). “Multisensory presence in virtual reality: possibilities & limitations,” in Multiple Sensorial Media Advances and Applications: New Developments in MulSeMedia. eds. Ghinea G. Andres F. Gulliver S. (Hershey: IGI Global), 1–38.

[ref51] Gallace A. Spence C. (2014). In Touch With the Future: The Sense of Touch From Cognitive Neuroscience to Virtual Reality. Oxford: Oxford University Press.

[ref52] Garcia M. (2016). Scents of place: airlines apply aromas for passenger comfort. *Apex News*, August 12. Available at: https://apex.aero/2016/08/12/airlines-apply-aromas-passenger-comfort (Accessed November 27, 2022).

[ref53] Garside J. (2004). Many more of us will work from home – or a cafe – says BT futurologist. *The Guardian*, January 2. Available at: https://www.theguardian.com/money/2014/jan/02/working-from-home-communications-technology-bt-futurologist (Accessed November 27, 2022).

[ref54] Gilmore J. H. Pine B. J. II (2002a). Differentiating hospitality operations via experiences: why selling services is not enough. Cornell Hotel Restaur. Adm. Q. 43, 87–96. doi: 10.1016/S0010-8804(02)80022-2

[ref55] Gilmore J. H. Pine B. J. II (2002b). Customer experience places: the new offering frontier. Strateg. Leadersh. 30, 4–11. doi: 10.1108/10878570210435306

[ref56] Goodin T. (2017). OFF. Your Digital Detox for a Better Life. London: Ilex Press.

[ref57] Gordon S. (2009). Smell of success: travel agents use holiday scents to sell trips. *Daily Mail Online*, June 29. Available at: https://www.dailymail.co.uk/travel/article-1196232/Holiday-smells-pumped-travel-agents-sell-trips.html (Accessed November 27, 2022).

[ref58] Gray W. (2019). Sensory pods and digital dining - is this wacky resort a sign of things to come? *The Daily Telegraph*, February 12. Available at: https://www.telegraph.co.uk/travel/destinations/europe/greece/articles/wellness-break-rhodes/ (Accessed November 27, 2022).

[ref59] Han H. Hyun S. (2014). Medical hotel in the growth of global medical tourism. J. Trav. Tour. Market. 31, 366–380. doi: 10.1080/10548408.2013.876955

[ref60] Hanefors M. Mossberg L. (2003). Searching for the extraordinary meal experience. J. Bus. Manag. 9, 249–270.

[ref61] Hardy M. Kirk-Smith M. D. Stretch D. D. (1995). Replacement of drug treatment for insomnia by ambient odor. Lancet 346:701. doi: 10.1016/S0140-6736(95)92310-1, PMID: 7658836

[ref62] Haslett S. (2019). Revealed: the tricks five-star hotels use to make rooms look perfect and polished – and how you can recreate them at home. *Daily Mail Online*, November 29. Available at: https://www.dailymail.co.uk/femail/article-7737253/The-tricks-five-star-hotels-use-look-polished-recreate-home.html (Accessed November 27, 2022).

[ref63] Heilig M. L. (1992). El cine del futuro: the cinema of the future. Presence 1, 279–294. doi: 10.1162/pres.1992.1.3.279

[ref64] Heschong L. (1979). Thermal Delight in Architecture. Cambridge, MA: MIT Press.

[ref65] Heung V. C. Gu T. (2012). Influence of restaurant atmospherics on patron satisfaction and behavioral intentions. Int. J. Hosp. Manag. 31, 1167–1177. doi: 10.1016/j.ijhm.2012.02.004

[ref66] Hill L. J. (2021). Joali being: inside the forthcoming Maldives resort dedicated to wellness. *Forbes*, June 15. Available at: https://www.forbes.com/sites/laurenjadehill/2021/06/15/joali-being-inside-the-forthcoming-maldives-resort-dedicated-to-wellness/?sh=3c992bc21196 (Accessed November 27, 2022).

[ref67] Hilton K. (2015). Psychology: the science of sensory marketing. Harv. Bus. Rev., 28–31. doi: 10.4324/978131569068126540780

[ref68] Holland R. W. Hendriks M. Aarts H. (2005). Smells like clean spirit. Nonconscious effects of scent on cognition and behavior. Psychol. Sci. 16, 689–693. doi: 10.1111/j.1467-9280.2005.01597.x, PMID: 16137254

[ref69] Hoppough S. (2006). What’s that smell? *Forbes*, September 15. Available at: https://www.forbes.com/forbes/2006/1002/076.html#3ef667ec5377 (Accessed November 27, 2022).

[ref70] Hou K. (2021). How baccarat rouge 540 became the scent of 2021. *The Cut*, December 21st. Available at: https://www.thecut.com/2021/12/how-baccarat-rouge-540-became-the-scent-of-2021.html (Accessed November 27, 2022).

[ref71] Howes D. (2014). “Introduction: “make it new!”—reforming the sensory world,” in A Cultural History of the Senses in the Modern Age. ed. Howes D. (London: Bloomsbury Academic), 2–30.

[ref72] Howes D. Classen C. (2014). Ways of Sensing: Understanding the Senses in Society. London: Routledge.

[ref73] Hunt E. (2016). Melbourne’s new ‘experience hotel’ lures millennials in the age of Airbnb. *The Guardian*, October 31. Available at: https://www.theguardian.com/travel/2016/nov/01/melbournes-new-experience-hotel-lures-millennials-in-the-age-of-airbnb (Accessed November 27, 2022).

[ref74] Hutmacher F. (2019). Why is there so much more research on vision than on any other sensory modality? Front. Psychol. 10:2246. doi: 10.3389/fpsyg.2019.02246, PMID: 31636589 PMC6787282

[ref75] Just M. G. Nichols L. M. Dunn R. R. (2019). Human indoor climate preferences approximate specific geographies. R. Soc. Open Sci. 6:180695. doi: 10.1098/rsos.180695, PMID: 31031985 PMC6458351

[ref76] Khan M. Garg R. J. Rahman J. (2015). Customer service experience in hotel operations: an empirical analysis. Procedia Soc. Behav. Sci. 189, 266–274. doi: 10.1016/j.sbspro.2015.03.222

[ref77] Khozaei F. Carbon C. C. Hosseini Nia M. Kim M. J. (2022). Preferences for hotels with biophilic design attributes in the post COVID-19 era. Buildings 12:427. doi: 10.3390/buildings12040427

[ref78] Kiatkawsin K. Han H. (2019). What drives customers’ willingness to pay price premiums for luxury gastronomic experiences at Michelin-starred restaurants? Int. J. Hosp. Manag. 82, 209–219. doi: 10.1016/j.ijhm.2019.04.024

[ref79] Kilikita J. (2018). A sensory wellness resort helped me manage my stress and anxiety. It’s all about tapping into those senses… *Elle*, April 21. Available at: https://www.elle.com/uk/life-and-culture/travel/a19879881/sensory-wellness-resort-stress-anxiety-tui-sensatori-negril-jamaica/ (Accessed November 27, 2022).

[ref80] Kim W. -H. Lee S. -H. Kim K. -S. (2020). Effects of sensory marketing on customer satisfaction and revisit intention in the hotel industry: the moderating roles of customers’ prior experience and gender. Anatolia 31, 523–535. doi: 10.1080/13032917.2020.1783692

[ref81] Kim D. Perdue R. R. (2013). The effects of cognitive, affective, and sensory attributes on hotel choice. Int. J. Hosp. Manag. 35, 246–257. doi: 10.1016/j.ijhm.2013.05.012

[ref82] Kingma B. van Marken Lichtenbelt W. D. (2015). Energy consumption in buildings and female thermal demand. Nat. Clim. Chang. 5, 1054–1056. doi: 10.1038/nclimate2741

[ref83] Kirk-Smith M. (2003). The psychological effects of lavender 1: in literature and plays. Int. J. Aromather. 13, 18–22. doi: 10.1016/S0962-4562(03)00046-8

[ref84] Kjellgren A. Buhrkall H. A. (2010). A comparison of the restorative effect of a natural environment with that of a simulated natural environment. J. Environ. Psychol. 30, 464–472. doi: 10.1016/j.jenvp.2010.01.011

[ref85] Knutson B. J. Beck J. A. Kim S. Cha J. (2009). Identifying the dimensions of the guest’s hotel experience. Cornell Hosp. Q. 50, 44–55. doi: 10.1177/1938965508326305

[ref86] Kotler P. (1974). Atmospherics as a marketing tool. J. Retail. 49, 48–64.

[ref87] Krieger M. H. (1973). What’s wrong with plastic trees? Artifice and authenticity in design. Science 179, 446–455. doi: 10.1126/science.179.4072.446, PMID: 17739132

[ref88] Krishna A. (2010). Sensory Marketing: Research on the Sensuality of Products. London: Routledge.

[ref89] Lampi E. (1973). Hotel and restaurant lighting. Cornell Hotel Restaur. Admin. Q. 13, 58–64. doi: 10.1177/001088047301300410

[ref90] Lanza J. (2004). Elevator Music: A Surreal History of Muzak, Easy-Listening, and Other Moodsong. Ann Arbor, MI: University of Michigan Press.

[ref91] LaTour K. A. Carbone L. P. (2014). Sticktion: assessing memory for the customer experience. Cornell Hosp. Q. 55, 342–353. doi: 10.1177/1938965514521689

[ref92] Lee M. Lee S. A. Koh Y. (2019). Multisensory experience for enhancing hotel guest experience. Int. J. Contemp. Hosp. Manag. 31, 4313–4337. doi: 10.1108/IJCHM-03-2018-0263

[ref93] Levere J. L. (2003). Skipping a night out on the town. *The New York Times*, February 25. Available at: http://www.nytimes.com/2003/02/25/business/business-travel-skipping-a-night-out-on-the-town.html?scp=38&sq=hotel%room,%20business%20traveller&st=cse (Accessed November 27, 2022).

[ref94] Li G. Law R. Vu H. Q. Rong J. Zhao X. R. (2015). Identifying emerging hotel preferences using emerging pattern mining technique. Tour. Manag. 46, 311–321. doi: 10.1016/j.tourman.2014.06.015

[ref95] Lindstrom M. (2005a). Brand Sense: How to Build Brands Through Touch, Taste, Smell, Sight and Sound. London: Kogan Page.

[ref96] Lindstrom M. (2005b). Broad sensory branding. J. Prod. Brand Manag. 14, 84–87. doi: 10.1108/10610420510592554

[ref97] Lundberg D. (1994). The Hotel and Restaurant Business, 6th Edn. New York, NY: Van Nostrand Reinhold.

[ref98] Magnini V. P. Parker E. E. (2009). The psychological effects of music: implications for hotel firms. J. Vacat. Mark. 15, 53–62. doi: 10.1177/1356766708098171

[ref99] Magnini V. P. Thelen S. T. (2008). The influence of music on perceptions of brand personality, décor, and service quality: the case of classical music in a fine-dining restaurant. J. Hosp. Leis. Mark. 16, 286–300. doi: 10.1080/10507050801946866

[ref100] Malefyt T. D. (2014). “An anthropology of the senses: tracing the future of sensory marketing in brand rituals,” in Handbook of Anthropology in Business. eds. Denny R. Sunderland P. (Walnut Creek, CA: Left Coast Press), 790–810.

[ref101] Malhotra N. K. (1984). Information and sensory overload. Information and sensory overload in psychology and marketing. Psychol. Mark. 1, 9–21. doi: 10.1002/mar.4220010304

[ref102] Marsan J. (1999). Ringing up revenues. *Hotels*, 33, September 9. Available at: http://www.hotelsmag.com/0999/0999tech.html (Accessed November 27, 2022).

[ref103] Martínez Polo L. (2022). Alimento futuro: Experiencia sensorial del hotel Click Clack. *El Tiempo*, July 16. Available at: https://www.eltiempo.com/cultura/gastronomia/alimento-futuro-una-cena-multisensorial-sobre-la-comida-del-manana-687857 (Accessed November 27, 2022).

[ref104] Masters B. (2022). Suite dreams. FT Weekend Magazine 11, 18–23.

[ref105] Matthews S. (2019). ‘Digital detox’ holidays where you ditch your phone let you enjoy your trip MORE ‘because you won’t be bombarded by notifications’. *Daily Mail Online*, August 14. Available at: https://www.dailymail.co.uk/health/article-7356493/Digital-detox-holidays-ditch-phone-make-enjoy-trip-more.html (Accessed November 27, 2022).

[ref106] Matthewson J. (2009). Today’s food newsbits: rouge tomate celebrates first year, first stat; Europe’s stars, food & art a success. *Daily Blender*, November 16. Available at: https://dailyblender.com/2009/11/todays-food-newsbits-rouge-tomate-celebrates-one-year-europes-stars-food-art-a-success/ (Accessed November 27, 2022).

[ref107] McGuire C. (2016). Would YOU think a plump receptionist is better at their job? Study shows guests give more positive reviews to hotels staffed by females with a larger frame. *Daily Mail Online*, January 27. Available at: https://www.dailymail.co.uk/travel/travel_news/article-3418975/Would-think-plump-receptionist-better-job-Study-shows-guests-positive-reviews-hotels-staffed-females-larger-frame.html (Accessed November 27, 2022).

[ref108] McGuire C. (2017). Heaven scent! Smelly flights could soon be a thing of the past thanks to perfume pumped into plane cabins *Daily Mail Online*, January 24. Available at: http://www.dailymail.co.uk/travel/travel_news/article4152042/whyairlinespumpingperfumeplanecabins.html (Accessed November 27, 2022).

[ref109] Meyer C. Schwager A. (2007). Understanding customer experience. Harv. Bus. Rev. 85, 116–126. 17345685

[ref110] Meyers-Levy J. Zhu R. J. (2007). The influence of ceiling height: the effect of priming on the type of processing that people use. J. Consum. Res. 34, 174–186. doi: 10.1086/519146

[ref111] Minsky L. Fahey C. Fabrigas C. (2018). Inside the invisible but influential world of scent branding. *Harvard Business Review*, April 11. Available at: https://hbr.org/2018/04/inside-the-invisible-but-influential-world-of-scent-branding (Accessed November 27, 2022).

[ref112] Montagu A. (1971). Touching: The Human Significance of the Skin. New York, NY: Columbia University Press.

[ref113] Morrin M. Chebat J. C. (2005). Person-place congruency: the interactive effects of shopper style and atmospherics on consumer expenditures. J. Serv. Res. 8, 181–191. doi: 10.1177/1094670505279420

[ref114] Nabham G. P. St. Antoine S. (1993). “The loss of floral and faunal story: the extinction of experience,” in The Biophilia Hypothesis. eds. Kellert S. R. Wilson E. O. (Washington, DC: Island Press), 229–250.

[ref115] Nichols W. J. (2014). Blue Mind: How Water Makes You Happier, More Connected and Better at What You Do. London, UK: Abacus.

[ref116] North A. C. Hargreaves D. J. McKendrick J. (1997). In-store music affects product choice. Nature 390:132. doi: 10.1038/36484

[ref117] North A. C. Hargreaves D. J. McKendrick J. (1999). The influence of in-store music on wine selections. J. Appl. Psychol. 84, 271–276. doi: 10.1037/0021-9010.84.2.271

[ref118] Nurminen M. (2003). *Guest Experience Design in Hotel Industry*. Master thesis submitted at the Faculty of Business Administration Management of technology. Canada: Simon Fraser University.

[ref119] Ogle A. (2009). Making sense of the hotel guestroom. J. Retail Leis. Prop. 8, 159–172. doi: 10.1057/rlp.2009.7

[ref120] Pacelle M. (1992). Many people refuse to check in if a hotel has odors in the lobby. *Wall Street Journal*, July 28, B1.

[ref121] Park J. Hadi R. (2020). Shivering for status: when cold temperatures increase product evaluation. J. Consum. Psychol. 30, 314–328. doi: 10.1002/jcpy.1133

[ref122] Park N. K. Pae J. Y. Meneely J. (2010). Cultural preferences in hotel guestroom lighting design. J. Inter. Des. 36, 21–34. doi: 10.1111/j.1939-1668.2010.01046.x

[ref123] Pelet J.-É. Lick E. Taieb B. (2021). The internet of things in upscale hotels: its impact on guests’ sensory experiences and behaviour. Int. J. Contemp. Hosp. Manag. 33, 4035–4056. doi: 10.1108/IJCHM-02-2021-0226

[ref124] Pine B. J. II Gilmore J. H. (1998). Welcome to the experience economy. Harv. Bus. Rev. 76, 97–105. 10181589

[ref125] Pine B. J. II Gilmore J. H. (1999). The Experience Economy: Work is Theatre & Every Business is a Stage. Boston, MA: Harvard Business Review Press.

[ref126] Postrel V. (2004). The Substance of Style: How the Rise of Aesthetic Value is Remaking Commerce, Culture, and Consciousness. New York, NY: Harper Perennial.

[ref127] Press Release. (2008). “*A-room-atherapy’ Helps Hotel Guests Nod Off*”. Press Release. June 17.

[ref128] Prochnik G. (2011). In Pursuit of Silence: Listening for Meaning in a World of Noise. New York, NY: Anchor.

[ref129] Robson D. (2022). How interior design choices can boost your mental and physical health. *New Scientist*, April 13. Available at: https://www.newscientist.com/article/mg25433823-000-how-interior-design-choices-can-boost-your-mental-and-physical-health/#ixzz7SccZ5mh7 (Accessed November 27, 2022).

[ref130] Rowley T. (2010). What do you fancy? Pizza express staff to be taught how to flirt with customers. *The Independent*, October 14, 24.

[ref131] Sabiniewicz A. Zimmermann P. Ozturk G. A. Warr J. Hummel T. (2022). Effects of odors on sleep quality in 139 healthy participants. Sci. Rep. 12:17165. doi: 10.1038/s41598-022-21371-5, PMID: 36229501 PMC9562345

[ref132] Salkin A. (2005). Shivering for luxury. *The New York Times*, June 26. Available at: https://www.nytimes.com/2005/06/26/fashion/sundaystyles/shivering-for-luxury.html (Accessed November 27, 2022).

[ref133] Sayin E. Krishna A. Ardelet C. Decré G. B. Goudey A. (2015). “Sound and safe,”: the effect of ambient sound on the perceived safety of public spaces. Int. J. Res. Mark. 32, 343–353. doi: 10.1016/j.ijresmar.2015.06.002

[ref134] Schifferstein H. N. J. (2006). The perceived importance of sensory modalities in product usage: a study of self-reports. Acta Psychol. 121, 41–64. doi: 10.1016/j.actpsy.2005.06.004, PMID: 16098945

[ref135] Schmitt B. H. (1999). Experiential Marketing: How to Get Customers to Sense, Feel, Think, Act and Relate to Your Company and Brand. New York, NY: Free Press.

[ref136] Shahid S. Paul J. (2022). Examining guests’ experience in luxury hotels: evidence from an emerging market. J. Mark. Manag., 1–29. doi: 10.1080/0267257X.2022.2085768

[ref137] Shahid S. Paul J. Gilal F. G. Ansari S. (2022). The role of sensory marketing and brand experience in building emotional attachment and brand loyalty in luxury retail stores. Psychol. Mark. 39, 1398–1412. doi: 10.1002/mar.21661

[ref138] Sharkey J. (2007). Building quieter hotels is a booming business. *The New York Times*, October 2. Available at: https://www.nytimes.com/2007/10/02/business/worldbusiness/02iht-noise.4.7720623.html (Accessed November 27, 2022).

[ref139] Shehata A. E. Alaswadi W. (2022). Can sensory marketing factors improve the customers’ pleasure and arousal in Egyptian resort hotels? J. Assoc. Arab Univ. Tour. Hosp. 22, 111–131. doi: 10.21608/jaauth.2022.120550.1296

[ref140] Shibata S. Suzuki N. (2004). Effect of an indoor plant on creative task performance and mood. Scand. J. Psychol. 45, 373–381. doi: 10.1111/j.1467-9450.2004.00419.x, PMID: 15535806

[ref141] Sklavos P. (2020). How hotels integrate sensory marketing techniques in their strategy. *Hotelier academy*, July 18. Available at: https://www.hotelieracademy.org/how-hotels-integrate-sensory-marketing-techniques-in-their-strategy/ (Accessed November 27, 2022).

[ref142] Spence C. (2002). The ICI Report on the Secret of the Senses. London: The Communication Group.

[ref143] Spence C. (2015a). Just how much of what we taste derives from the sense of smell? Flavour 4:30. doi: 10.1186/s13411-015-0040-2

[ref144] Spence C. (2015b). Leading the consumer by the nose: on the commercialization of olfactory-design for the food and beverage sector. Flavour 4:31. doi: 10.1186/s13411-015-0041-1

[ref145] Spence C. (2016). Gastrodiplomacy: assessing the role of food in decision-making. Flavour 5:4. doi: 10.1186/s13411-016-0050-8

[ref146] Spence C. (2017). Gastrophysics: The New Science of Eating. London: Viking Penguin.

[ref147] Spence C. (2020a). Senses of space: designing for the multisensory mind. Cogn. Res. 5:46. doi: 10.1186/s41235-020-00243-4 PMC7501350 32945978

[ref148] Spence C. (2020b). Temperature-based crossmodal correspondences: Causes & consequences. Multisens. Res. 33, 645–682. doi: 10.1163/22134808-20191494, PMID: 31923885

[ref149] Spence C. (2020c). Using ambient scent to enhance well-being in the multisensory built environment. Front. Psychol. 11:598859. doi: 10.3389/fpsyg.2020.598859, PMID: 33329267 PMC7710513

[ref150] Spence C. (2020d). “Atmospheric effects on eating and drinking: a review,” in Handbook of Eating and Drinking. ed. Meiselman H. (Cham: Springer), 257–276.

[ref151] Spence C. (2021a). Scent in motion: on the multiple uses of ambient scent in the context of passenger transport. Front. Psychol. 12:702517. doi: 10.3389/fpsyg.2021.702517, PMID: 34322070 PMC8312487

[ref152] Spence C. (2021b). Sensehacking: How to Use the Power of Your Senses for Happier, Heathier Living. London: Viking Penguin.

[ref153] Spence C. (2021c). Explaining seasonal patterns of food consumption. Int. J. Gastron. Food Sci. 24:100332. doi: 10.1016/j.ijgfs.2021.100332

[ref154] Spence C. (2022a). Multisensory contributions to affective touch. Curr. Opin. Behav. Sci. 43, 40–45. doi: 10.1016/j.cobeha.2021.08.003

[ref155] Spence C. (2022b). On the use of ambient odours to influence the multisensory experience of dining. Int. J. Gastron. Food Sci. 27:100444. doi: 10.1016/j.ijgfs.2021.100444

[ref156] Spence C. Okajima K. Cheok A. D. Petit O. Michel C. (2016). Eating with our eyes: from visual hunger to digital satiation. Brain Cogn. 110, 53–63. doi: 10.1016/j.bandc.2015.08.006, PMID: 26432045

[ref157] Spence C. Piqueras-Fiszman B. (2012). Dining in the dark: why, exactly, is the experience so popular? The Psychologist 25, 888–891.

[ref158] Spence C. Piqueras-Fiszman B. (2014). The Perfect Meal: The Multisensory Science of Food and Dining. Oxford: Wiley-Blackwell.

[ref159] Spence C. Reinoso-Carvalho F. Velasco C. Wang Q. J. (2019). Extrinsic auditory contributions to food perception & consumer behaviour: an interdisciplinary review. Multisens. Res. 32, 275–318. doi: 10.1163/22134808-20191403, PMID: 31059484

[ref160] Spiegel A. (2014). Visiting a restaurant? There’s a good chance it’s googling you. *The Huffington Post*, April 14. Available at: http://www.huffingtonpost.com/2014/04/14/restaurants-googling-patrons_n_5132535.html (Accessed November 27, 2022).

[ref161] Stead S. Wetzels R. Wetzels M. Odekerken-Schröder G. Mahr D. (2022). Toward multisensory customer experiences: a cross-disciplinary bibliometric review and future research directions. J. Serv. Res. 25, 440–459. doi: 10.1177/10946705221079941

[ref162] Stephens M. (2013). Noise is most common complaint in online hotel reviews. *eHotelier*, September 29. Available at: https://insights.ehotelier.com/news/2013/09/29/noise-is-most-common-complaint-in-online-hotel-reviews/ (Accessed November 27, 2022).

[ref163] Sterne J. (1997). Sounds like the mall of America: programmed music and the architectonics of commercial space. Ethnomusicology 41, 22–50. doi: 10.2307/852577

[ref164] Strøm-Tejsen P. Zukowska D. Wargocki P. Wyon D. P. (2016). The effects of bedroom air quality on sleep and next-day performance. Indoor Air 26, 679–686. doi: 10.1111/ina.12254, PMID: 26452168

[ref165] Suh M. Moon H. Han H. Ham S. (2015). Invisible and intangible, but undeniable: role of ambient conditions in building hotel guests’ loyalty. J. Hosp. Mark. Manag. 24, 727–753. doi: 10.1080/19368623.2014.945223

[ref166] Tamaki M. Won Bang J. Watanabe T. Sasaki Y. (2016). Night watch in one brain hemisphere during sleep associated with the first-night effect in humans. Curr. Biol. 26, 1190–1194. doi: 10.1016/j.cub.2016.02.063, PMID: 27112296 PMC4864126

[ref167] Thomson N. H. (2010). *Virtual experiential marketing at Marriott International, Inc.: an examination of effects on consumer purchase intentions*. Cameron School of Business. University of North Carolina Wilmington

[ref168] Thornhill T. (2015). The secret psychology of hotel design: how guests’ moods and desires are changed by the look and layout of the building. *Daily Mail Online*, October 13. Available at: https://www.dailymail.co.uk/travel/travel_news/article-3266888/The-secret-psychology-hotel-design-guests-moods-desires-changed-look-layout-building.html (Accessed November 27, 2022).

[ref169] Thornhill T. (2022). Is this New York’s most exquisite (and nicest-smelling) hotel? Inside the Baccarat, which drips with crystal chandeliers and is scented with one of Rihanna’s favourite perfumes. *Daily Mail Online*, April 10. Available at: https://www.dailymail.co.uk/travel/escape/article-10669587/Is-New-Yorks-exquisite-nicest-smelling-hotel-review-114-room-Baccarat.html (Accessed November 27, 2022).

[ref170] TMI Spy Hotels and Leisure (2015). Winter.

[ref171] Trompeta M. A. Karantinou K. Koritos C. Bijmolt T. H. A. (2022). A meta-analysis of the effects of music in tourism and hospitality settings. J. Bus. Res. 138, 130–145. doi: 10.1016/j.jbusres.2021.08.067

[ref172] Tsushima Y. Okada S. Kawai Y. Sumita A. Ando H. Miki M. (2020). Effect of illumination on perceived temperature. PLoS One 15:e0236321. doi: 10.1371/journal.pone.0236321, PMID: 32776987 PMC7416916

[ref173] Tui (2018). Luxury holiday concept Tui Sensatori celebrates 10 years of sense fuelled holidays as new Rhodes resort gets ready for opening. February 20. Available at: https://www.tui.co.uk/press/luxury-holiday-concept-tui-sensatori-celebrates-10-years-sense-fuelled-holidays-new-rhodes-resort-gets-ready-opening/ (Accessed November 27, 2022).

[ref174] Vardarı L. Arapı D. (2017). Experimental marketing in hotel operations. Bingöl Üniversitesi Sosyal Bilimler Enstitüsü Dergisi (BUSBED) 7, 177–192. doi: 10.29029/busbed.348950

[ref175] Vartanian O. Navarrete G. Chatterjee A. Fich L. B. Leder H. Modroño C.. (2013). Impact of contour on aesthetic judgments and approach-avoidance decisions in architecture. Proc. Nat. Acad. Sci. U.S.A. 110, 10446–10453. doi: 10.1073/pnas.1301227110 PMC3690611 23754408

[ref176] Vickers G. Spence C. (2007). Get set for the sensory side of the century. *Contact: Royal Mail’s Magazine for Marketers*, November, 11–14.

[ref177] Villa-Clarke A. (2020). Smells like innovation: how the peninsula hotels is reinventing its in-house beauty products. *Forbes*, November 18. Available at: https://www.forbes.com/sites/angelinavillaclarke/2020/11/18/smells-like-innovation-how-the-peninsula-hotels-is-reinventing-its-in-house-beauty-products/?sh=2d1c72294508 (Accessed November 27, 2022).

[ref178] Walker R. (2004). The hidden (in plain sight) persuaders. *The New York Times Magazine*, December 5, 68–75. https://www.nytimes.com/2004/12/05/magazine/the-hidden-in-plain-sight-persuaders.html (Accessed November 27, 2022).

[ref179] Walls A. R. (2013). A cross-sectional examination of hotel consumer experience and relative effects on consumer values. Int. J. Hosp. Manag. 32, 179–192. doi: 10.1016/j.ijhm.2012.04.009

[ref180] Walls A. Okumus F. Wang R. Y. Kwun D. J.-W. (2011). Understanding the consumer experience: an exploratory study of luxury hotels. J. Hosp. Market. Manag. 20, 166–197. doi: 10.1080/19368623.2011.536074

[ref181] Whitelocks S. (2018). Would the sound of a dishwasher get you to sleep? easyHotel launches a lullaby service for guests to make them feel more at home. *Daily Mail Online*, May 14. Available at: http://www.dailymail.co.uk/travel/travel_news/article-5726093/easyHotel-launches-lullaby-service-guests-make-feel-home.html (Accessed November 27, 2022).

[ref182] Wiedmann K. P. Hennigs N. Klarmann C. Behrens S. (2013). Creating multi-sensory experiences in luxury marketing. Mark. Rev. St. Gallen 30, 60–69. doi: 10.1365/s11621-013-0300-4

[ref183] Wiedmann K.-P. Labenz F. Haase J. Hennigs N. (2016). Soothe your senses: a multisensory approach to customer experience management and value creation in luxury tourism. *European Business Review*, January-February, 50–55. Available at: https://www.europeanbusinessreview.com/soothe-your-senses/ (Accessed November 27, 2022).

[ref184] Wiedmann K. P. Labenz F. Haase J. Hennigs N. (2018). The power of experiential marketing: exploring the causal relationships among multisensory marketing, brand experience, customer perceived value and brand strength. J. Brand Manag. 25, 101–118. doi: 10.1057/s41262-017-0061-5

[ref185] Willander J. Larsson M. (2007). Olfaction and emotion: the case of autobiographical memory. Mem. Cogn. 35, 1659–1663. doi: 10.3758/BF03193499, PMID: 18062543

[ref186] Williams F. (2017). The Nature Fix: Why Nature Makes Us Happier, Healthier, and More Creative. London: W. W. Norton & Company.

[ref187] Wober M. (1966). Sensotypes. J. Soc. Psychol. 70, 181–189. doi: 10.1080/00224545.1966.9712414, PMID: 5980219

[ref188] Wober M. (1991). “The sensotype hypothesis” in The Varieties of Sensory Experience: A Sourcebook in the Anthropology of the Senses. ed. Howes D. (Toronto: University of Toronto Press), 31–42.

[ref189] Wood D. (2019). The top 5 hotel guest complaints and how staff can respond. *eHotelier*, January 9. Available at: https://insights.ehotelier.com/insights/2019/01/09/the-top-5-hotel-guest-complaints-and-how-staff-can-respond/ (Accessed November 27, 2022).

[ref190] Wörfel P. Frentz F. Tautu C. (2022). Marketing comes to its senses: a bibliometric review and integrated framework of sensory experience in marketing. Eur. J. Mark. 56, 704–737. doi: 10.1108/EJM-07-2020-0510

[ref191] Wu C. H. J. Liang R. D. (2009). Effect of experiential value on customer satisfaction with service encounters in luxury-hotel restaurants. Int. J. Hosp. Manag. 28, 586–593. doi: 10.1016/j.ijhm.2009.03.008

[ref192] Xu J. B. Chan A. (2010). A conceptual framework of hotel experience and customer-based brand equity: some research questions and implications. Int. J. Contemp. Hosp. Manag. 22, 174–193. doi: 10.1108/09596111011018179

[ref193] Yu C.-E. Xie S. Y. Wen J. (2020). Coloring the destination: the role of color psychology on Instagram. Tour. Manag. 80:104110. doi: 10.1016/j.tourman.2020.104110

[ref194] Zaldivar G. (2017). Hotel guests’ most common complaints. *Travel Pulse*, July 3rd. Available at: https://www.travelpulse.com/news/hotels-and-resorts/hotel-guests-most-common-complaints.html (Accessed November 27, 2022).

[ref195] Zellner D. Geller T. Lyons S. Pyper A. Riaz K. (2017). Ethnic congruence of music and food affects food selection but not liking. Food Qual. Prefer. 56, 126–129. doi: 10.1016/j.foodqual.2016.10.004

[ref196] Zemke D. M. Shoemaker S. (2007). Scent across the crowded room: exploring the effect of ambient scent on social interactions. Hosp. Manag. 26, 927–940. doi: 10.1016/j.ijhm.2006.10.009

[ref197] Zemke D. M. Shoemaker S. (2008). A sociable atmosphere: ambient scent’s effect on social interaction. Cornell Hosp. Q. 49, 317–329. doi: 10.1177/1938965508320626

[ref198] Zha D. Foroudi P. Jin Z. Melewar T. C. (2022). Making sense of sensory brand experience: constructing an integrative framework for future research. Int. J. Manag. Rev. 24, 130–167. doi: 10.1111/ijmr.12270

[ref199] Zhu R. Argo J. J. (2013). Exploring the impact of various shaped seating arrangements on persuasion. J. Consum. Res. 40, 336–349. doi: 10.1086/670392

